# Brain-derived neurotrophic factor differentially modulates excitability of two classes of hippocampal output neurons

**DOI:** 10.1152/jn.00186.2016

**Published:** 2016-05-04

**Authors:** A. R. Graves, S. J. Moore, N. Spruston, A. K. Tryba, C. C. Kaczorowski

**Affiliations:** ^1^Department of Neurobiology, Northwestern University, Evanston, Illinois;; ^2^Howard Hughes Medical Institute, Janelia Research Campus, Ashburn, Virginia;; ^3^Molecular and Behavioral Neuroscience Institute, University of Michigan, Ann Arbor, Michigan;; ^4^Department of Physiology, Medical College of Wisconsin, Milwaukee, Wisconsin;; ^5^Department of Pediatrics, The University of Chicago, Chicago, Illinois; and; ^6^Department of Anatomy and Neurobiology, University of Tennessee Health Science Center, Memphis, Tennessee

**Keywords:** hippocampus, metabotropic glutamate receptors, burst firing, intrinsic plasticity, long-term potentiation

## Abstract

Brain-derived neurotrophic factor (BDNF) plays an important role in hippocampus-dependent learning and memory. Canonically, this has been ascribed to an enhancing effect on neuronal excitability and synaptic plasticity in the CA1 region. However, it is the pyramidal neurons in the subiculum that form the primary efferent pathways conveying hippocampal information to other areas of the brain, and yet the effect of BDNF on these neurons has remained unexplored. We present new data that BDNF regulates neuronal excitability and cellular plasticity in a much more complex manner than previously suggested. Subicular pyramidal neurons can be divided into two major classes, which have different electrophysiological and morphological properties, different requirements for the induction of plasticity, and different extrahippocampal projections. We found that BDNF increases excitability in one class of subicular pyramidal neurons yet decreases excitability in the other class. Furthermore, while endogenous BDNF was necessary for the induction of synaptic plasticity in both cell types, BDNF enhanced intrinsic plasticity in one class of pyramidal neurons yet suppressed intrinsic plasticity in the other. Taken together, these data suggest a novel role for BDNF signaling, as it appears to dynamically and bidirectionally regulate the output of hippocampal information to different regions of the brain.

## Abstract (precis)

*We show that BDNF mediates acute and long-lasting changes in intrinsic neuronal excitability in two classes of subicular pyramidal neurons (EB and LB neurons). Although BDNF plays similar roles in the induction of synaptic plasticity in these two cell types, it differentially and bidirectionally affects intrinsic excitability and burst plasticity in EB and LB neurons. These cell type-specific effects represent new avenues by which BDNF can influence the well-established information storage function of the hippocampus*.

## NEW & NOTEWORTHY

*We show that BDNF mediates acute and long-lasting changes in intrinsic neuronal excitability in two classes of subicular pyramidal neurons (EB and LB neurons). Although BDNF plays similar roles in the induction of synaptic plasticity in these two cell types, it differentially and bidirectionally affects intrinsic excitability and burst plasticity in EB and LB neurons. These cell type-specific effects represent new avenues by which BDNF can influence the well-established information storage function of the hippocampus*.

brain-derived neurotrophic factor (BDNF) is proposed to play a critical role in hippocampus-dependent learning and memory tasks. Indeed, in animal models the acquisition of spatial memory tasks (e.g., Morris water maze and contextual fear conditioning) increases the expression of BDNF within the hippocampus, while interfering with BDNF signaling (genetically or pharmacologically) impairs performance on these memory tasks (for review, see Lu et al. 2008). Similarly, in humans dysregulation of BDNF in the hippocampus is proposed to underlie memory and cognitive disorders (Sanchez et al. 2011; Tapia-Arancibia et al. 2008). The role of BDNF in mediating hippocampus-dependent memory has been largely attributed to modulation of synaptic plasticity in the form of long-term potentiation (LTP), a leading candidate mechanism underlying information storage. Stimulation patterns that induce LTP increase BDNF expression within the hippocampus (Patterson et al. 1992), and exogenous BDNF application facilitates LTP (Figurov et al. 1996; Kang and Schuman 1995; Korte et al. 1995; Patterson et al. 1996), whereas stimulation patterns leading to long-term depression are accompanied by a reduction in BDNF (Aicardi et al. 2004). Additionally, decreasing BDNF signaling through the TrkB receptor impairs LTP (Lu et al. 2008).

The major output pathway of the hippocampus is formed by pyramidal neurons of the subiculum that convey information to downstream target brain areas. Lesions of this region have been shown to disrupt performance on hippocampus-dependent learning and memory tasks (Bindu et al. 2005). There are two distinct classes of pyramidal neurons in the subiculum, and they differ in their electrophysiological characteristics, morphology, connectivity with other brain regions, and specific requirements for the induction of both synaptic and nonsynaptic (or intrinsic) plasticity (Behr et al. 2009; Graves et al. 2012; Kim and Spruston 2012; Menendez de la Prida et al. 2003; Menendez de la Prida and Gal 2004; Moore et al. 2009; Stewart and Wong 1993; Wozny et al. 2008). Thus these two classes of neurons likely have distinct roles in processing different modalities of information in the brain.

Here we investigated the role of BDNF in regulating neuronal excitability, synaptic plasticity, and intrinsic plasticity in the two classes of subicular pyramidal neurons. Our results show that BDNF signaling is required for the induction of synaptic plasticity in both neuron types, while bidirectionally altering acute neuronal excitability and long-lasting intrinsic plasticity in these two populations. Taken together, these data demonstrate a novel role for BDNF signaling in modulating neuronal function in this critical brain region and suggest that BDNF signaling may act as a switch to direct the flow of hippocampally processed information to specific downstream regions to facilitate the encoding and storage of long-term memories.

## MATERIALS AND METHODS

Male mice were colony-housed (12:12-h light-dark cycle) with ad libitum food and water in accordance with protocols approved by the Animal Care and Use Committees at the Medical College of Wisconsin, Northwestern University, and University of Tennessee Health Science Center. Mice at P20–P28 were anesthetized and hippocampal slices prepared as previously described (Neuner et al. 2015). Briefly, the mouse was deeply anesthetized under isoflurane and decapitated, and the brain was quickly removed and placed into ice-cold artificial cerebrospinal fluid (ACSF) containing (mM) 125 NaCl, 25 glucose, 25 NaHCO_3_, 2.5 KCl, 1.25 NaH_2_PO_4_, 2 CaCl_2_, 1 MgCl_2_; pH 7.5, bubbled with 95% O_2_-5% CO_2_. A blocking cut was made to obtain transverse slices that maintained intact subiculum apical dendrites within the hippocampal slice. Slices (400 μm) of the medial hippocampus and adjacent cortex were made with a Leica vibratome (Wetzlar, Germany). The slices were incubated at 34°C in oxygenated ACSF for 30 min and then maintained at room temperature (22°C) for 1–4 h before use.

### 

#### Drugs and solutions.

A standard K-gluconate internal solution was used (Neuner et al. 2015). Where indicated, recombinant human BDNF (50 ng/ml; Neuromics, Edina, MN) was bath applied. Other bath-applied drugs included ionotropic glutamate receptor (iGluR) antagonists CNQX (20 μM) and CPP (20 μM); metabotropic glutamate receptor (mGluR) antagonists LY367385 (25 μM) and MPEP (10 μM) from Tocris-Cookson (Bristol, UK); and either the BDNF scavenger TrkB-Fc or control IgG (1 μg/ml) (Rex et al. 2007).

#### Electrophysiological recordings.

Neuronal firing properties and plasticity were evaluated by somatic whole cell current-clamp recordings from subicular pyramidal neurons (3- to 5-MΩ open-tip resistance) at 30–33°C. Neurons were held at −67 mV (unless measuring resting membrane potentials; Table 1). Series resistance and capacitance were monitored and compensated throughout recordings; neurons with >40-MΩ series resistance were excluded. Early-bursting (EB) neurons exhibited a burst of two or more action potentials (occurring at >100 Hz), while late-bursting (LB) neurons (which have also been referred to as “regular-firing neurons”) exhibited a single action potential. Neuronal excitability was quantified by injecting a long-lasting depolarizing current step (2 s, 100 pA). Synaptic responses were evoked with an extracellular glass stimulating pipette placed 50–200 μm away from the site of the whole cell recording on the apical dendritic side of the soma. Intrinsically driven burst firing was assessed by a train of 10 excitatory postsynaptic current (EPSC)-like somatic current injections (0.8–2.0 nA), the amplitude of which was initially set so that, for each train, approximately four responses were bursts of two action potentials while the remaining six responses consisted of single action potentials (Graves et al. 2012). Once set, the amplitude of this current injection remained unchanged for the duration of the experiment. We then monitored excitatory postsynaptic potentials (EPSPs) and intrinsically driven burst firing events across a 10-min baseline period, which was initiated within ∼5 min of whole cell configuration. To induce both synaptic and intrinsic plasticity, a theta-burst stimulation (TBS) protocol was used that consisted of theta-patterned synaptic activation (5 stimuli at 100 Hz) of proximal neuronal inputs paired with a brief (2 ms) somatic current injection (at the burst-monitoring amplitude), repeated at 5 Hz for 3 s. Both power analyses and replication of prior study results were used to confirm that sample sizes were appropriate to yield sufficient statistical power. Statistical analyses of group data were performed with Prism (GraphPad software, La Jolla, CA) and SigmaPlot (Systat Software, San Jose, CA). All analyses were corrected for multiple comparisons. Unless stated otherwise, reported values are means ± SE. Cells that required >200 pA of holding current to maintain these potentials were excluded from the data set, and treatments were randomized.

**Table 1.** Effects of BDNF on membrane properties of subicular neurons

	*R*_N_, MΩ	*V*_rest_, mV	*V*_thr_, mV	FWHM, ms
Early bursting				
ACSF	94 ± 17	−68.3 ± 1.8	−44.2 ± 1.7	0.76 ± 0.1
BDNF	80 ± 16	−61.2 ± 1.4*	−49.0 ± 1.4*	0.75 ± 0.1
Late bursting				
ACSF	116 ± 21	−71.3 ± 1.3	−48.6 ± 1.3	0.75 ± 0.1
BDNF	116 ± 22	−70.4 ± 1.0	−48.2 ± 0.6	0.80 ± 0.1*

Values are means ± SE. Input resistance (*R*_N_), resting membrane potential (*V*_rest_), action potential threshold (*V*_thr_), and spike width (FWHM) were measured for late-bursting (LB) and early-bursting (EB) neurons in normal ACSF and after 10-min exposure to exogenous BDNF.

* Significantly different from ACSF, *P* < 0.05.

## RESULTS

### 

#### Differential effect of BDNF on intrinsic excitability of distinct classes of subicular neurons.

Neurons were classified on the basis of their firing response to a brief (2 ms) depolarizing current injection. Similar to studies in rats (Jarsky et al. 2008), nearly half of the neurons in the subiculum of mice fired bursts of two or more spikes in response to current injections just above threshold (41%; 31 of 75). These neurons have been referred to as burst-firing, but more recently classified as early-bursting (EB), neurons because they generate bursts at the beginning of a train of short current pulses (Graves et al. 2012). The remaining neurons (59%; 44 of 75) fired single action potentials with threshold-level current injections but could be driven to burst by increasing the current injection amplitude. Neurons of this type have been referred to as regular-firing, but more recently classified as late-bursting (LB), neurons because they generate bursts toward the end of a train of short current pulses (Graves et al. 2012). Throughout this report, the terms EB and LB are used to distinguish between these two types of neurons.

**Fig. 1.**
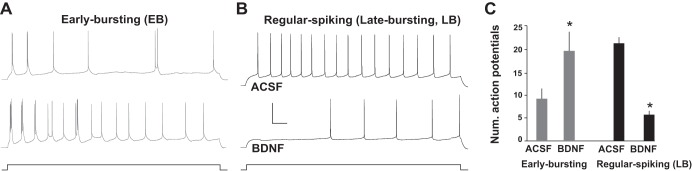
BDNF differentially affects firing properties of subicular neurons. *A* and *B*: step-current injections (100 pA, 2 s) were used to evoke firing in normal ACSF and in the presence of BDNF (50 ng/ml). Exposure to BDNF for 10 min significantly increased firing in early-bursting (EB) neurons (*A*) but significantly reduced firing in late-bursting (LB) neurons (*B*). Scale bar, 40 mV and 100 ms. *C*: summary plot showing dichotomy of BDNF-mediated changes in the excitability of EB vs. LB neurons evidenced by a significant difference in number of action potentials elicited with long current steps in these neurons. *Significantly different from ACSF, *P* < 0.05.

The effect of acute BDNF on EB and LB neuronal excitability was quantified by comparing the total number of action potentials elicited by a depolarizing current step (2 s, 100 pA) before and after bath application of BDNF (10 min, 50 ng/ml). Interestingly, BDNF had opposite effects on the excitability of EB and LB neurons: BDNF increased EB firing but decreased firing in LB neurons (Fig. 1). It was previously suggested that exogenous BDNF (at 50 ng/ml) increases spontaneous firing rates in hippocampal neurons via potentiation of excitatory transmission and receptor-mediated depolarization (Levine et al. 1995). To rule out synaptic effects, we replicated these findings in the presence of iGluR antagonists (20 μM CNQX and 20 μM CPP); BDNF increased EB neuron excitability (iGluR antagonists = 4.0 ± 2.1 vs. BDNF = 8.4 ± 4.2 spikes; *n* = 10; paired *t*-test, *P* < 0.05) and decreased excitability of LB neurons (iGluR antagonists = 12.9 ± 2.9 vs. BDNF = 6.5 ± 2.15 spikes; *n* = 11; paired *t*-test, *P* < 0.001). These data indicate that exogenous BDNF differentially affects excitability of EB and LB neurons by modulating intrinsic neuronal properties.

#### BDNF is required for synaptic plasticity in both EB and LB subicular neurons.

**Fig. 2.**
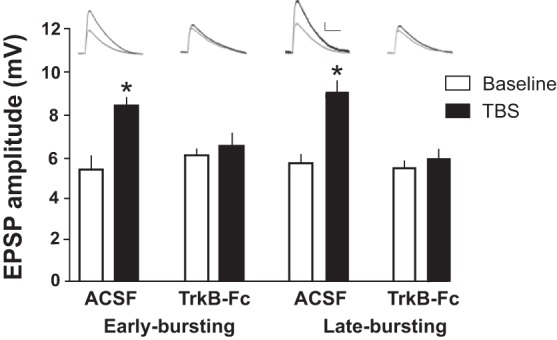
Chelating synaptically released BDNF blocks the induction of LTP. In both EB and LB neurons, LTP was induced with TBS in normal ACSF but was prevented in the presence of the high-affinity BDNF chelator TrkB-Fc. Representative traces (gray, baseline; black, after TBS) are shown for ACSF and TrkB-Fc conditions. Scale bar, 2 mV and 50 ms. Analysis of variance revealed a significant effect of TrkB-Fc treatment on EPSP amplitude after TBS [*F*(1,23) = 11.3, *P* < 0.0001] that was independent of cell type [*F*(1,23) = 0.15, *P* = 0.71]. *Significantly different from Baseline, *P* < 0.05.

While BDNF has been shown to regulate synaptic plasticity at Schaffer collateral synapses on CA1 pyramidal neurons (Cunha et al. 2010), the effect of BDNF on plasticity mechanisms in the subiculum has not been explored. To investigate the role of BDNF in mediating synaptic plasticity, we monitored EPSPs in subicular pyramidal neurons during a 10-min baseline period and 30–40 min after TBS in either the absence or the presence of the BDNF scavenger TrkB-Fc. In ACSF alone, TBS induced LTP in both EB and LB neurons measured at 30–40 min after TBS relative to baseline (EB_baseline_ = 5.44 ± 0.33 vs. EB_TBS_ = 8.54 ± 0.34 and LB_baseline_ = 5.76 ± 0.19 vs. LB_TBS_ = 9.17 ± 0.37; *n* = 3/group), similar to an earlier study (Fig. 2) (Behr et al. 2009). However, when TrkB-Fc was present, synaptic plasticity was prevented in EB and LB neurons (EB-TrkB-Fc_baseline_ = 6.16 ± 0.28 vs. EB-TrkB-Fc_TBS_ = 6.67 ± 0.30 and LB-TrkB-Fc_baseline_ = 5.50 ± 0.29 vs. LBTrkB-Fc_TBS_ = 6.00 ± 0.33; *n* = 3/group), indicating that BDNF signaling is required for LTP in subicular neurons (Fig. 2). Analysis of variance revealed a significant effect of TrkB-Fc treatment on EPSP amplitude after TBS [*F*(1,23) = 11.3, *P* < 0.0001] that was independent of cell type [*F*(1,23) = 0.15, *P* = 0.71]. These data are consistent with previous work showing that TrkB-Fc chelation of BDNF prevents LTP of Schaffer collateral-CA1 synapses after TBS (Figurov et al. 1996; Kang et al. 1997; Rex et al. 2007).

#### BDNF has opposite effects on intrinsic plasticity of burst firing in EB and LB neurons.

In addition to synaptic plasticity, EB and LB neurons also exhibit a form of intrinsic plasticity that results in a change in the propensity for burst firing (i.e., burst plasticity; Graves et al. 2012; Moore et al. 2009). Interestingly, the burst plasticity is bidirectional (both an increase and a decrease in burst firing can be induced, depending on conditions) and displays a cell type-specific dependence on activation of mGluRs. Given our new observation that BDNF has divergent effects on intrinsic excitability, we hypothesized that BDNF signaling may also differentially regulate induction of burst plasticity in EB and LB neurons.

**Fig. 3.**
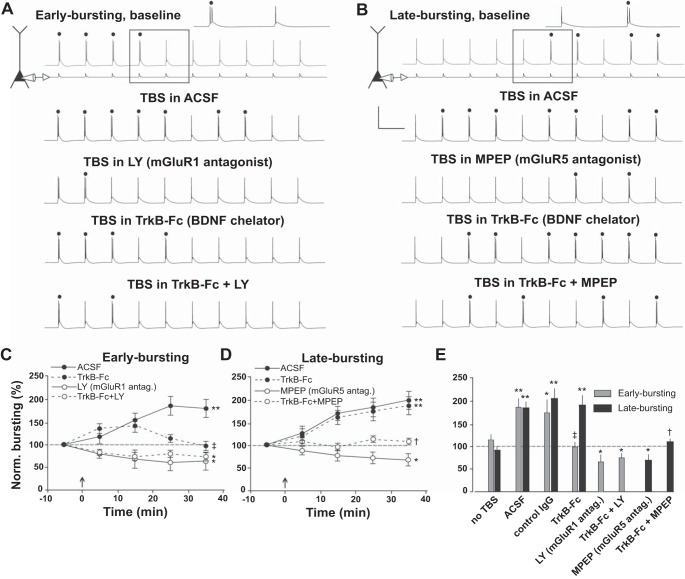
Cell-specific modulation of intrinsic plasticity by BDNF. *A* and *B*: representative traces depict burst firing patterns of EB and LB neurons. Bursts are denoted by dots and were evoked with a train of 10 individual EPSC-like current injections at 5 Hz, the amplitude of which was sufficient to evoke 4 bursts per train of 10 inputs during the baseline period (*top*, gray traces). *Inset*: magnified representation of burst and single spike for each neuron class. Note that bursts occurred early in the train in EB neurons and later in the train in LB neurons. Representative traces of bursting patterns with the same stimulus after TBS in several experimental conditions are shown in black (*A* and *B*, *bottom* 4 traces). *C–E*: group data for control and experimental conditions are presented for both cell types and are normalized to pre-TBS baseline levels of burst firing. Asterisks indicate a significant difference from baseline: *P < 0.05, **P < 0.01. Note that chelating synaptically released BDNF with TrkB-Fc selectively prevents the induction of burst enhancement in EB neurons [*C*; ‡*t*(1,8) = 0.02, *P* = 0.9] and the induction of burst suppression in LB neurons [*D*; †*t*(1,8) = 2.36, *P* = 0.18]. *E*: summary of effect (average normalized burst firing 30–40 min after TBS) of TrkB-Fc and mGluR antagonists on burst enhancement and suppression. LY, LY367385.

Neuronal output was monitored with a train of 10 EPSC-like somatic current injections at an amplitude and a frequency sufficient to evoke four burst responses per train (the remaining 6 responses were single action potentials; top gray traces in Fig. 3, *A* and *B*). As previously reported in rats (Graves et al. 2012; Moore et al. 2009), TBS delivered in control conditions (ACSF) induced a long-lasting increase in the number of burst responses in both EB and LB neurons (*n* = 5/cell type, *P* < 0.0001) in mouse subiculum (Fig. 3). In contrast, a long-lasting decrease in the number of burst responses was induced when TBS was delivered in the presence of blockers of different types of mGluRs (Fig. 3): an mGluR1 antagonist (LY367385; *n* = 4, *P* < 0.01) for EB neurons and an mGluR5 antagonist (MPEP *n* = 4, *P* < 0.007) for LB neurons. These results replicate prior work by Graves et al. (2012). Importantly, both the increase and decrease in burst firing were activity dependent, because no change was observed in the absence of TBS in either neuronal population (Fig. 3*E*).

To determine whether endogenous BDNF is necessary for the induction of burst plasticity in EB or LB neurons, we bath-applied the BDNF scavenger TrkB-Fc for 30 min before delivering TBS in the cell type-specific conditions that normally (i.e., without blocking BDNF signaling) result in either enhancement or suppression of burst firing. Interestingly, in EB neurons, chelating BDNF with TrkB-Fc prevented the long-lasting increase in burst firing following TBS [*t*(1,8) = 0.02, *P* = 0.9] but did not block the decrease in burst firing following blockade of mGluR1 (Fig. 3, *A*, *C*, *E*) [*t*(1,8) = 15.1, *P* < 0.008]. Conversely, in LB neurons, TrkB-Fc had no effect on the TBS-induced increase in burst firing [*t*(1,8) = 29.9, *P* < 0.0006] but instead completely prevented the expected decrease in burst firing that results when mGluR5 are blocked [*t*(1,8) = 2.36, *P* = 0.18] (Fig. 3, *B*, *D*, *E*). Importantly, these cell type-specific effects of TrkB-Fc demonstrate that BDNF modulates burst plasticity of subicular pyramidal neurons in a bidirectional manner that depends on distinct signaling mechanisms in each class of pyramidal neuron.

## DISCUSSION

Our study demonstrates that BDNF regulates synaptic plasticity and mediates both acute and long-lasting changes in intrinsic neuronal excitability in two classes of subicular pyramidal neurons (EB and LB neurons). Interestingly, though BDNF plays a similar role in the induction of synaptic plasticity in these two cell types, it differentially and bidirectionally affects intrinsic excitability and burst plasticity in EB and LB neurons. The cell type-specific effects identified in this study represent new avenues by which BDNF can influence the well-established information storage function of the hippocampus.

### 

#### BDNF modulates neuronal firing via intrinsic mechanisms.

Previous studies showed that exogenous BDNF can increase firing of hippocampal neurons in vitro by enhancing neurotransmission (for review, see Cunha et al. 2010). Similarly, exogenous BDNF in dorsal horn neurons has been shown to differentially affect action potential firing in distinct cell types through effects on synaptic efficacy (Lu et al. 2007). Importantly, in our experiments the effect of BDNF on neuronal excitability in both EB and LB neurons persisted in the presence of iGluR antagonists, which demonstrates for the first time that BDNF can regulate neuronal excitability directly via modulation of intrinsic ionic conductances.

#### BDNF modulates plasticity of intrinsic excitability in hippocampal neurons.

In addition to synaptic plasticity (Bliss and Collingridge 1993), an alternative mechanism that may contribute to learning and memory is intrinsic plasticity, which refers to long-lasting changes in nonsynaptic (or intrinsic) conductances. Although many types of intrinsic plasticity have been described, modulation of these mechanisms by endogenous signaling molecules like BDNF has remained largely unexplored. To investigate the role of BDNF in regulating the induction of intrinsic plasticity, we took advantage of a particular form of intrinsic plasticity, called burst plasticity, that has been characterized in EB and LB subicular pyramidal neurons (Graves et al. 2012; Moore et al. 2009). Our experiments show that BDNF signaling is required for burst plasticity in both EB and LB neurons, but interestingly this regulation is cell type specific: in EB neurons BDNF signaling was only necessary to induce an increase in burst firing (but not a decrease), while conversely in LB neurons BDNF signaling was only necessary to induce a decrease in burst firing (but not an increase). These data demonstrate that BDNF plays a novel role in mediating intrinsic burst plasticity by altering intrinsic conductances to change neuronal activity.

#### Implications for information processing, learning and memory, and disease.

The subiculum is the primary output pathway of the hippocampus, with efferent fibers targeting several cortical and subcortical regions. Importantly, EB and LB neurons exhibit a significant divergence in their projections (Kim and Spruston 2012) and are also thought to carry different types of spatial information (primarily global vs. local cues) (Knierim et al. 2014). As a result, altering the function of these subicular neurons is likely to strongly influence the gating of hippocampally processed information to different brain regions.

BDNF is a canonical member of the neurotrophin family that has well-described signaling pathways and mechanisms (Huang and Reichardt 2003; Segal 2003; Segal and Greenberg 1996). Binding of BDNF to its cognate receptor, trkB, initiates receptor dimerization and reciprocal phosphorylation of each receptor at tyrosine residues. In addition to increasing catalytic activity, phosphorylation of specific tyrosine residues results in recruitment and binding of signaling molecules that can activate three intracellular signaling cascades: *1*) the phosphatidylinositol 3-kinase/Akt pathway, which is generally thought to be critical for neuronal survival; *2*) the phospholipase C pathway, which catalyzes the breakdown of lipids into diacylglycerol, resulting in activation of protein kinase C, and inositol 1,4,5-trisphosphate, resulting in an increase in Ca^2+^ release from intracellular stores; and *3*) the Ras/MAPK (microtubule-associated protein kinase) cascade, which can phosphorylate and thereby activate or alter the activity of myriad proteins (including additional kinases); furthermore, the Ras/MAPK pathway can also effect changes in transcription via activation of transcription factors including cAMP response element binding protein (CREB). Some or all of these various signaling cascades may be differentially involved in the cell type-specific effects of BDNF on burst plasticity, which may include differences in the pathways recruited, differences in the cellular compartments in which these pathways are activated, or even interaction with additional pathways or signaling molecular that have yet to be elucidated.

Interestingly, BDNF has been shown to affect hippocampal function in both a constitutive and an activity-dependent manner (Cunha et al. 2010). Constitutive BDNF expression in the adult hippocampus has classically been ascribed a trophic effect, regulating the survival of neurons and maintenance of appropriate anatomical connections (Huang and Reichardt 2001). However, physiological conditions that result in the global elevation of BDNF in the adult hippocampus have been shown to facilitate spatial memory (Griffin et al. 2009), suggesting that an elevation of the constitutive level of BDNF may enhance hippocampal processing and/or relay of spatial information. The effects on neuronal excitability that we describe may contribute to this facilitation of spatial memory, as increases in exogenous BDNF act as a bidirectional switch to privilege hippocampally processed information by upregulating output from EB cells (conveying the spatial context of an experience) while downregulating output from LB cells (conveying local content within an experience) (Knierim et al. 2014) (Fig. 1). Thus these cell type-specific effects of BDNF may engage different brain regions (e.g., an increase in BDNF in the subiculum may enhance output of spatial information to regions preferentially targeted by EB neurons and reduce LB-mediated throughput of information to nonspatial targets). On the other hand, for targets where EB and LB projections converge, constitutive expression of BDNF may increase the overall gain of neuronal output from the hippocampus by increasing burst firing properties of EB neurons, which is posited to enhance the fidelity of information transfer (Lisman 1997; Pike et al. 1999), thereby possibly enhancing fidelity of hippocampally processed spatial information.

On the other hand, activity-dependent release of BDNF (e.g., during theta-rhythmic activity, TBS) is thought to facilitate hippocampus-dependent learning and memory by enhancing synaptic plasticity (Cunha et al. 2010). Consistent with this model, our experiments show that BDNF signaling is necessary for induction of LTP in both EB and LB neurons (Fig. 2). In addition, we demonstrate that BDNF is involved in the induction of intrinsic plasticity, which is posited as a complementary cellular mechanism of learning and memory. In this case, the effects of activity-dependent release of BDNF accompanied by local neurotransmitter release and activation of synaptic receptors including mGluRs are more complex. We show that mGluR1 activation and BDNF signaling are necessary to enhance the output of EB neurons after TBS, whereas TBS enhances the output of LB cells in the absence of BDNF signaling (Fig. 3*E*, TrkB-Fc). Previous work has shown that these two cell types are inversely modulated by synergistic activation of mGluR1 and metabotropic acetylcholine receptors (Graves et al. 2012). Taken together with our results, these data suggest that BDNF may also operate in concert with mGluR1 to differentially modulate plasticity of burst firing in EB and LB subicular neurons, providing additional mechanisms whereby BDNF regulates behavioral flexibility (Sakata et al. 2013). Taken together, our data showing cell type-specific modulation of hippocampal output neurons suggests new mechanisms by which BDNF may contribute to learning and memory.

## GRANTS

A. R. Graves was supported by National Institutes of Health (NIH) Grants F31 NS-067758 and T32 MH-067564. A. R. Graves and N. Spruston were supported by NIH Grant RO1 NS-35180. A. K. Tryba was supported by an Advancing Healthier Wisconsin grant to A. K. Tryba. C. C. Kaczorowski was supported by NIH Grant K99/R00 AG-039511.

## DISCLOSURES

No conflicts of interest, financial or otherwise, are declared by the author(s).

## AUTHOR CONTRIBUTIONS

A.R.G., S.J.M., N.S., A.K.T., and C.C.K. conception and design of research; A.R.G. and C.C.K. performed experiments; A.R.G., S.J.M., and C.C.K. analyzed data; A.R.G., S.J.M., N.S., A.K.T., and C.C.K. interpreted results of experiments; A.R.G., S.J.M., and C.C.K. prepared figures; A.R.G., S.J.M., N.S., A.K.T., and C.C.K. edited and revised manuscript; A.R.G., S.J.M., N.S., A.K.T., and C.C.K. approved final version of manuscript; S.J.M. and C.C.K. drafted manuscript.
